# Cultural-Religious Approach: An Effective Community-Based Disaster Management Strategy for Reducing the Mortality and Morbidity of the Fourth Wave of Coronavirus Pandemic Caused by the Lineage B.1.1.7 (the British Variant) in Iran (Spring 2021)

**DOI:** 10.21315/mjms2022.29.1.16

**Published:** 2022-02-23

**Authors:** Hossein Ghaedamini, Hamid Jafari, Salman Farahbakhsh, Hesan Arefi, Zahra Saghafi

**Affiliations:** 1 Geriatric Care Research Center, Rafsanjan University of Medical Sciences, Rafsanjan, Iran; 2 Department of Medical Emergencies, Sirjan School of Medical Sciences, Serjan, Iran; 3 Department of Occupational Health, School of Medical Sciences, Sirjan Faculty of Medical Sciences, Serjan, Iran; 4 Kerman University of Medical Sciences, Kerman, Iran; 5 School of Nursing and Midwifery, Geriatric Care Research Center, Rafsanjan University of Medical Sciences, Rafsanjan, Iran

**Keywords:** COVID-19, Iran, community-based disaster management

## Abstract

Lineage B.1.1.7 (the British variant) is a new variant of SARS-CoV-2. The virus was first identified in the UK in October 2020. Since Iran is one of the most disaster risk countries in the world, disaster management is one of the most important issues. One of the effective approaches of this field is community-based disaster management (CBDM). Altogether, planning and policy-making through using various cultural-religious role models with emphasis on the cultural points can be useful to reduce the mortality and morbidity rate caused by the fourth wave of coronavirus in Iran.

Dear Editor,

Lineage B.1.1.7 (the British variant) is a new variant of SARS-CoV-2. The virus was first identified in the UK in October 2020. This variant has 17 mutations. The prevalence and mortality of this variant are quintuple and triple higher, respectively (1, 2).

There have been three waves of coronavirus in Iran (February–March 2020, May–June 2020 and September–October 2020) and the following has accelerated the fourth wave of coronavirus:

- spread of the lineage B.1.1.7 outbreak (January 2021)
- reducing people’s attention to related health instructions
- lack of sufficient vaccine for all people

The beginning of the Persian New Year, (which begins on the spring equinox and it is celebrated on 20 March 2021) is the most important cultural challenge. At this time, due to the New Year holidays, the amount of shopping (especially in big cities like Tehran, Isfahan, Mashhad, Shiraz and Tabriz), travels and, New Year celebrations and family parties increased significantly, it accelerated the spread of the coronavirus (3, 4).

Last year (March 2020), Iran had similar conditions, but this year, due to the prevalence of lineage B.1.1.7 and the difficulty to diagnose, higher infection rate, higher severity of this variant, and infections in children and adolescents (in addition to other age groups), the mortality and morbidity rate will increase significantly (5, 6).

Since Iran is one of the most disaster risk countries in the world, disaster management is one of the most important issues. One of the effective approaches of this field is the community-based disaster management (CBDM) (7).

The interaction of culture and religion in Iran (in line with the CBDM) has a significant impact on the management of various disasters (8). We believe approaching based on two factors (culture and religion) can play an effective role in reducing the mortality and morbidity of the fourth peak in Iran.

On the other hand, the use of a religious-cultural approach can be associated with challenges such as profound lack of trust in some officials about the effectiveness of cultural concepts and lack of acceptability among people about the benefits of religious concepts, interference of governments’ tasks, and religious institutions’ duties, inappropriate public awareness and allocating insufficient funding (9, 10).

To reduce the challenges of the religious-cultural approach, we believe that the use of various religious-cultural role models is an effective way to influence specific groups of society. These role models include the following:

- religious leaders (supreme leader, source of authorities) target group: religious people in the community
- prominent scientists and researchers of the country target group: students and university professors
- favourite artists, actors and athletes target group: most of the young people
- national role models target group: all members of society
- motivational psychologists and speakers target group: all members of society

Altogether, planning and policy-making through using various cultural-religious role models (in line with CBDM) with emphasis on the following points can be useful to reduce the mortality and morbidity rate caused by the fourth wave of coronavirus in Iran:

- strengthening the motivation of people for observing mandatory quarantine instructions, observing the social distance, using standard masks and having good hand hygiene
- encouraging people to get vaccinated
- cooperation with medical staff and other government agencies
- reducing travels, New Year (Nowruz) shopping (shopping through a virtual platform), ceremonies and parties (holding ceremonies virtually)
- providing appropriate programmes to increase knowledge about lineage B.1.1.7 in the form of smartphone applications, podcasts and audiobooks
- providing ‘take care’ packages for low incomes families
- providing facilities to families who follow health protocols related to coronavirus pandemic
